# Supplementary figures and images for: A Hepatitis C virus genotype 1b post-transplant isolate with high replication efficiency in cell culture and its adaptation to infectious virus production in vitro and in vivo
Source: PLoS Pathog. 2022 Jun 28;18(6):e1010472. doi: 10.1371/journal.ppat.1010472 (PMC9273080; doi:10.1371/journal.ppat.1010472)

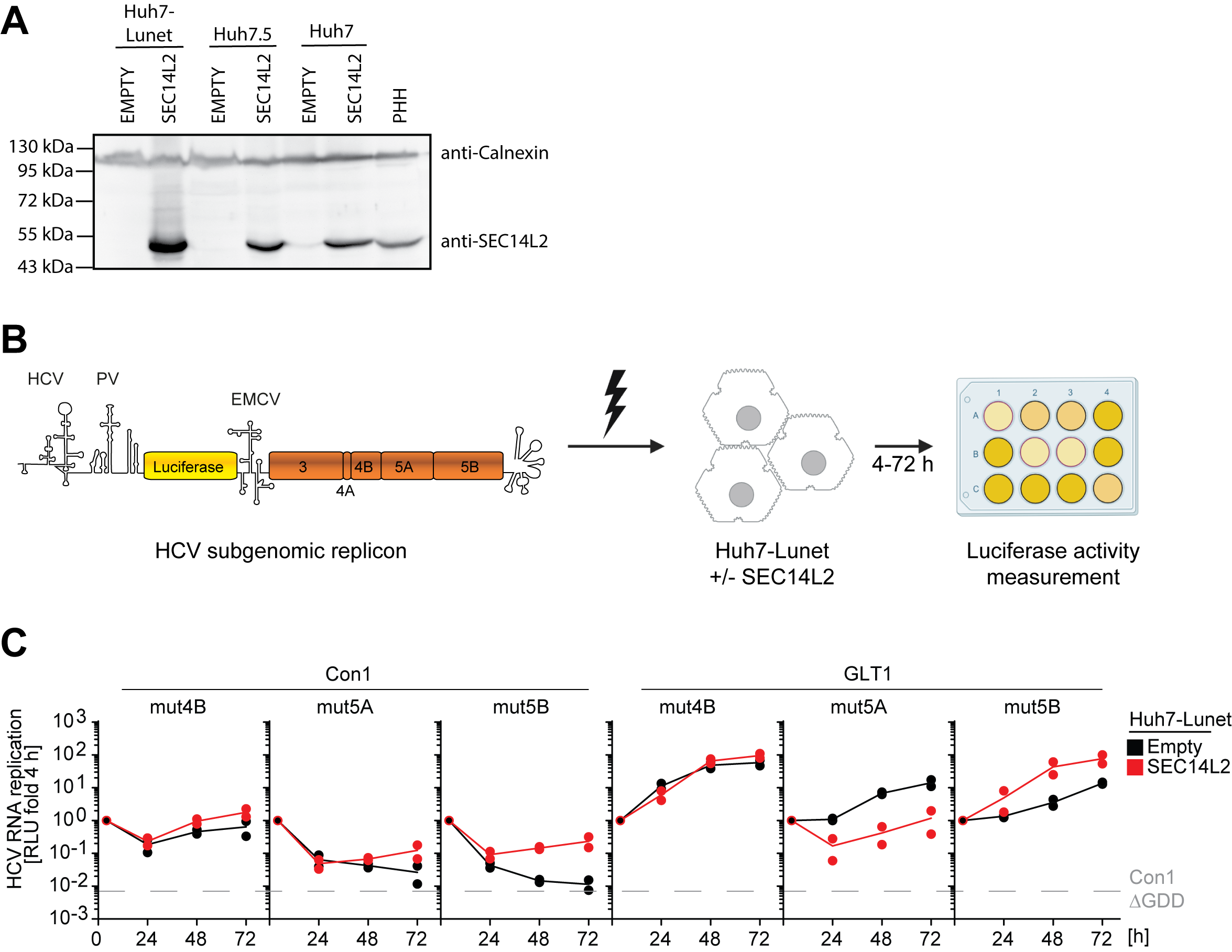

Supplement: S1 Fig — (A) SEC14L2 expression levels in Huh7-Lunet, Huh7.5 and Huh7 cells after lentiviral transduction with a SEC14L2 encoding vector or empty control, compared to primary human hepatocytes (PHH). Approximately 1x10^5^ Huh7-Lunet, Huh7.5, Huh7 or PHH were lysed and analyzed by 10% SDS-PAGE/Western blotting for SEC14L2 and Calnexin expression. One representative experiment from two independent repetitions is shown. (B) Schematic of a subgenomic reporter replicon and of the experimental procedure of luciferase-based replication measurement. HCV 5’UTR (HCV), poliovirus internal ribosomal entry site (IRES) (PV) and EMCV-IRES (EMCV), as well as the HCV 3’UTR are indicated by their respective secondary structures. NS3-NS5B and firefly luciferase coding regions are indicated by orange and yellow boxes, respectively. Transfection by electroporation is visualized by a lightning-symbol. (C) Replication enhancement by a combination of mutations and SEC14L2 expression for Con1 (left) and GLT1 (right). Huh7-Lunet cells either expressing SEC14L2 or transduced with empty vector were electroporated with the indicated subgenomic reporter replicon RNA containing mutations in NS4B (K1846T), NS5A (S2204R) or NS5B (R2884G) as indicated. Luciferase activity in cell lysates (RLU) was quantified as a correlate of RNA replication efficiency at the given time points and normalized to 4 h. A replication deficient Con1 variant (Con1ΔGDD) was used as a negative control and the respective luciferase level at 72 h is indicated by a dashed grey line in all diagrams. The data are the mean values from two independent experiments shown as individual data points with two technical replicates each. (TIF) [file ppat.1010472.s001.tif]

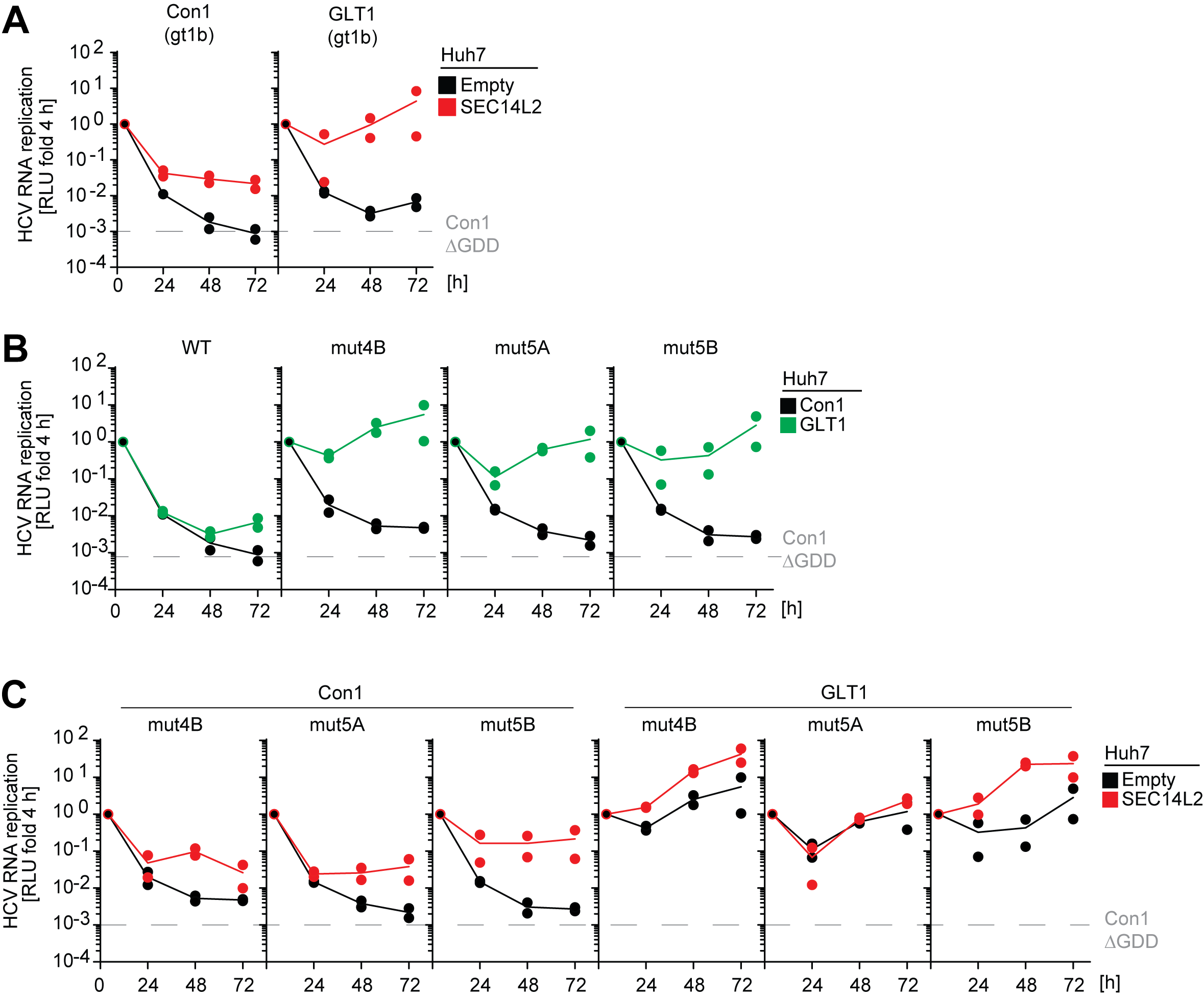

Supplement: S2 Fig — Huh7 cells were transfected with subgenomic reporter replicons of the indicated isolates or mutants. Luciferase activity in cell lysates (RLU) was quantified as a correlate of RNA replication efficiency at the given time points and normalized to 4 h. (A,C) HCV replication was stimulated by SEC14L2 expression compared to empty vector transduction as indicated. (B,C) Replication enhancement of GLT1 (green lines) or Con1 (black lines) by mutations in NS4B (K1846T), NS5A (S2204R) or NS5B (R2884G). A replication deficient Con1 variant (Con1ΔGDD) was used as a negative control for replication and the respective luciferase level at 72 h is indicated by a dashed grey line in all diagrams. The data are the mean values from two independent experiments shown as individual data points with two technical replicates each. (TIF) [file ppat.1010472.s002.tif]

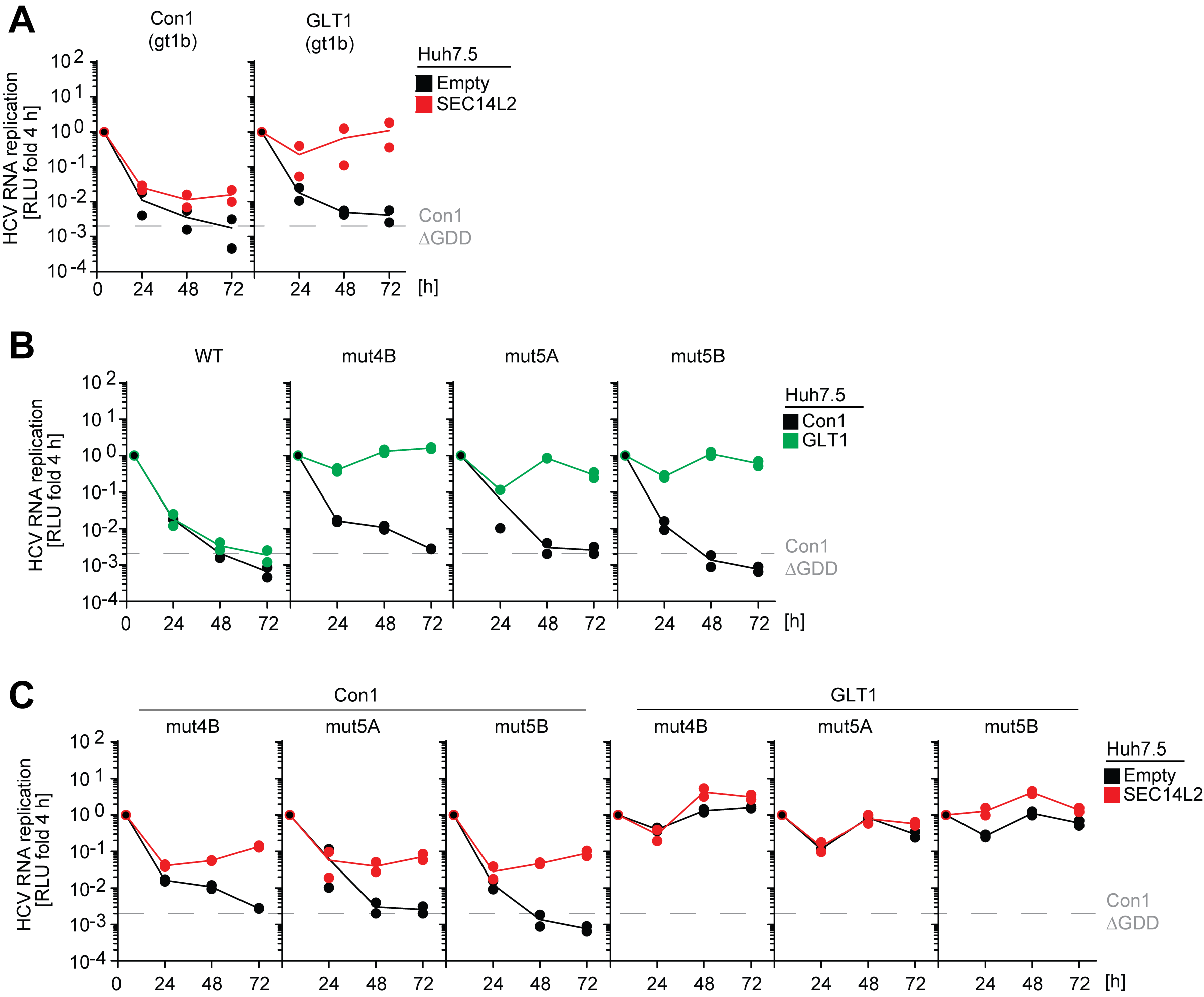

Supplement: S3 Fig — Huh7.5 cells were transfected with subgenomic reporter replicons of the indicated isolates or mutants. Luciferase activity in cell lysates (RLU) was quantified as a correlate of RNA replication efficiency at the given time points and normalized to 4 h. (A,C) HCV replication was stimulated by SEC14L2 expression compared to empty vector transduction as indicated. (B,C) Replication enhancement of GLT1 (green lines) or Con1 (black lines) by mutations in NS4B (K1846T), NS5A (S2204R) or NS5B (R2884G). A replication deficient Con1 variant (Con1ΔGDD) was used as a negative control for replication and the respective luciferase level at 72 h is indicated by a dashed grey line in all diagrams. The data are the mean values from two independent experiments shown as individual data points with two technical replicates each. (TIF) [file ppat.1010472.s003.tif]

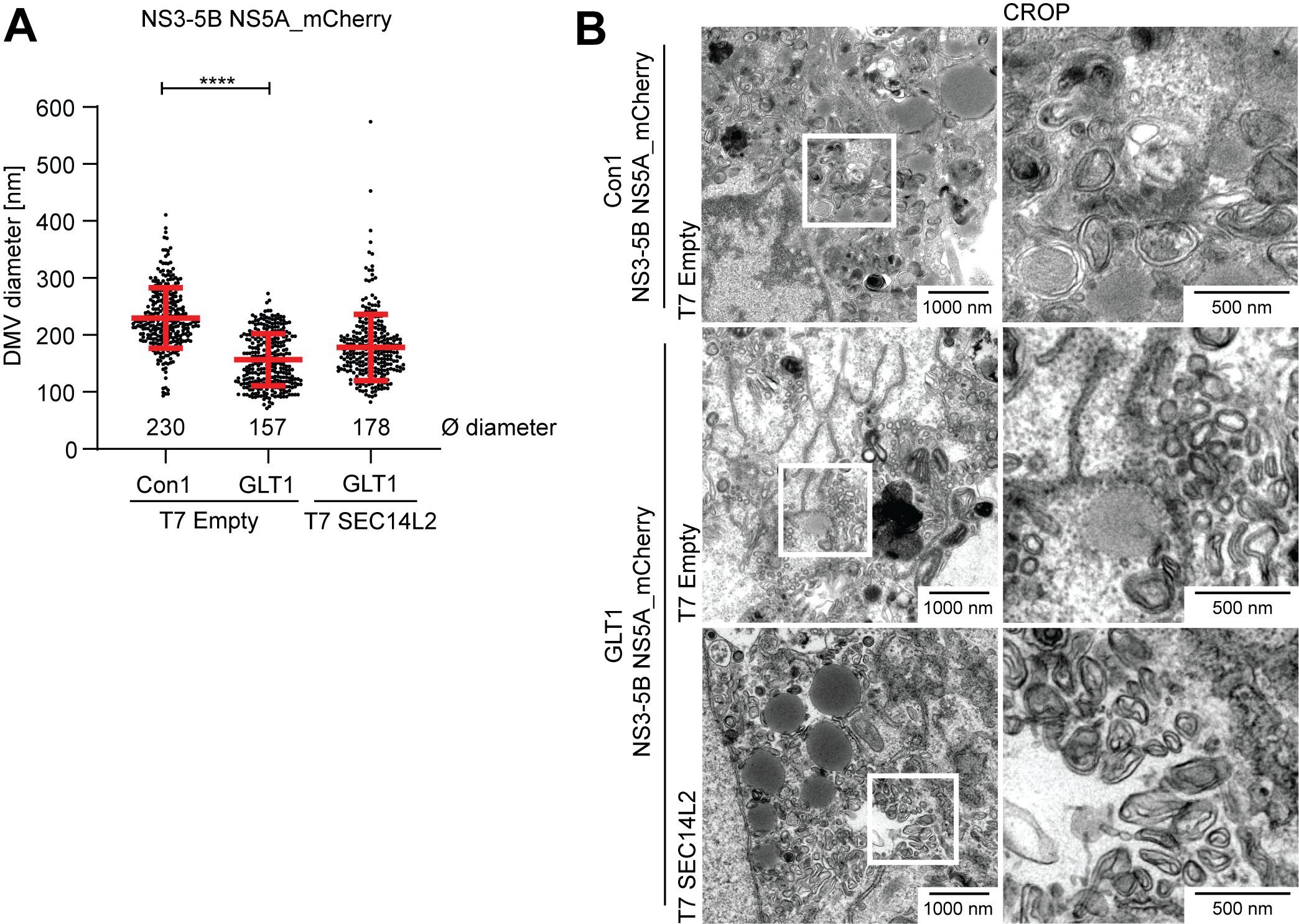

Supplement: S4 Fig — Huh7-Lunet T7 cells either expressing SEC14L2 (T7 SEC14L2) or not (T7 empty) were transfected with a pTM vector encoding either Con1 or GLT1 NS3-5B with an in frame insertion of mCherry in domain 3 of NS5A [72]. Cells were fixed 24 hours post transfection and the mCherry fluorescent signal was used to identify positive cells for further analysis by CLEM. (A) A minimum of six cells were analyzed and the DMV diameter was measured. Unpaired two-tailed Student’s t-test was used to determine statistical significance (**** = p ≤ 0.0001). (B) Representative images of each condition. (TIF) [file ppat.1010472.s004.tif]

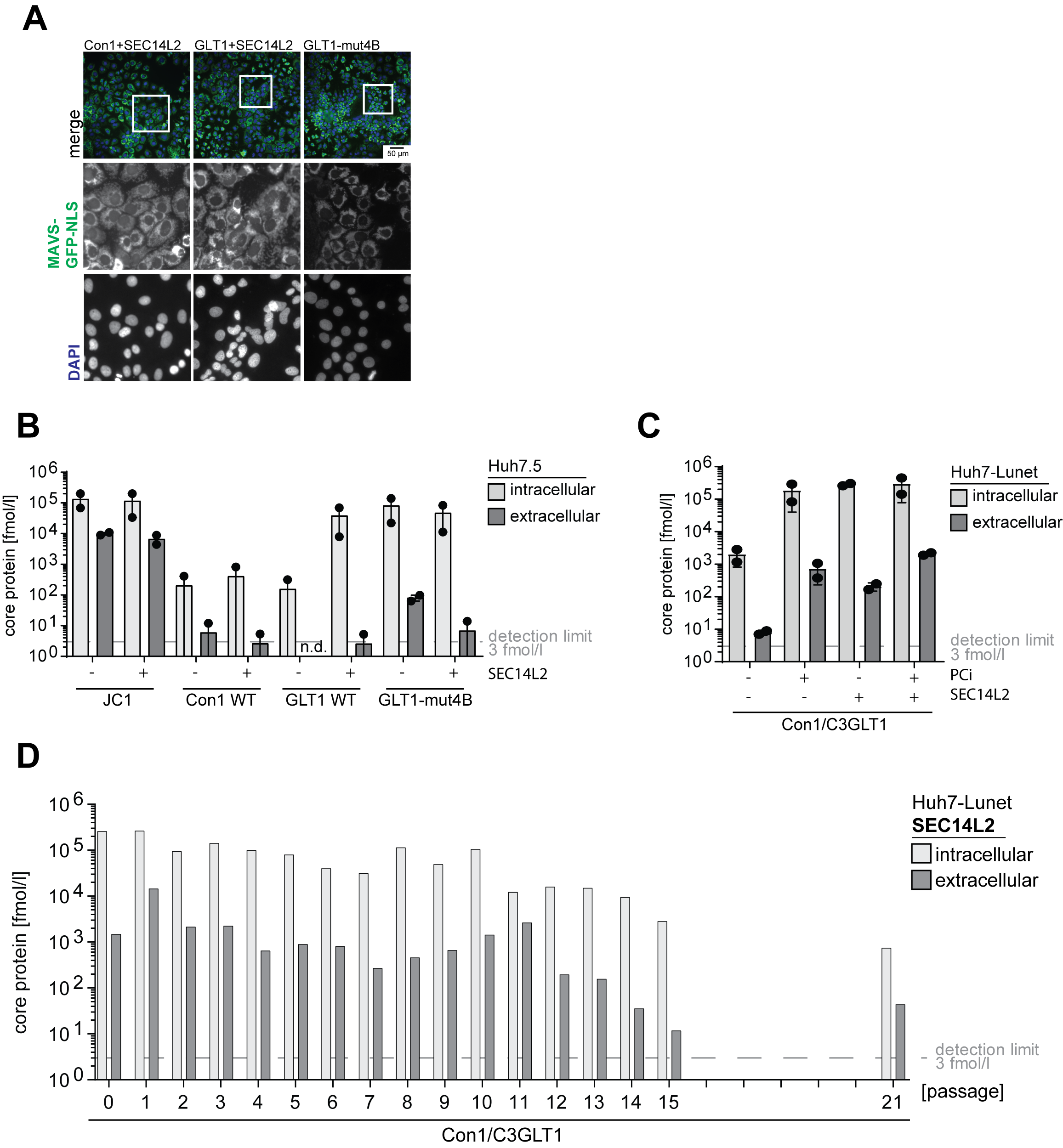

Supplement: S5 Fig — (A) Examples of lack of infection events of indicated isolates upon supernatant transfer to MAVS-GFP-NLS cells. Infection is identified by nuclear GFP signal. (B) Detection of intra- and extracellular core protein after transfection of full-length virus genomes. Huh7.5 cells with or without SEC14L2 expression were transfected with the indicated HCV full-length genomes and intra- and extracellular core protein levels were determined by ELISA as correlates of replication and virus secretion, respectively. (C,D) A chimeric GLT1 genome, encoding the structural proteins of Con1 up to the C3 junction site in NS2 [25] was transfected in Huh7-Lunet (C) or Huh7-Lunet CD81 (D) cells with or without SEC14L2 expression and/or PCi treatment. Intra- and extracellular core levels were determined by Elisa 72 hours post transfection (C) or before passaging (D). Shown are data from two independent experiments (B, C) or from a single passaging experiment (D). The dashed grey line indicates the detection limit of 3 fmol/l core protein; n.d. = not detectable. (TIF) [file ppat.1010472.s005.tif]

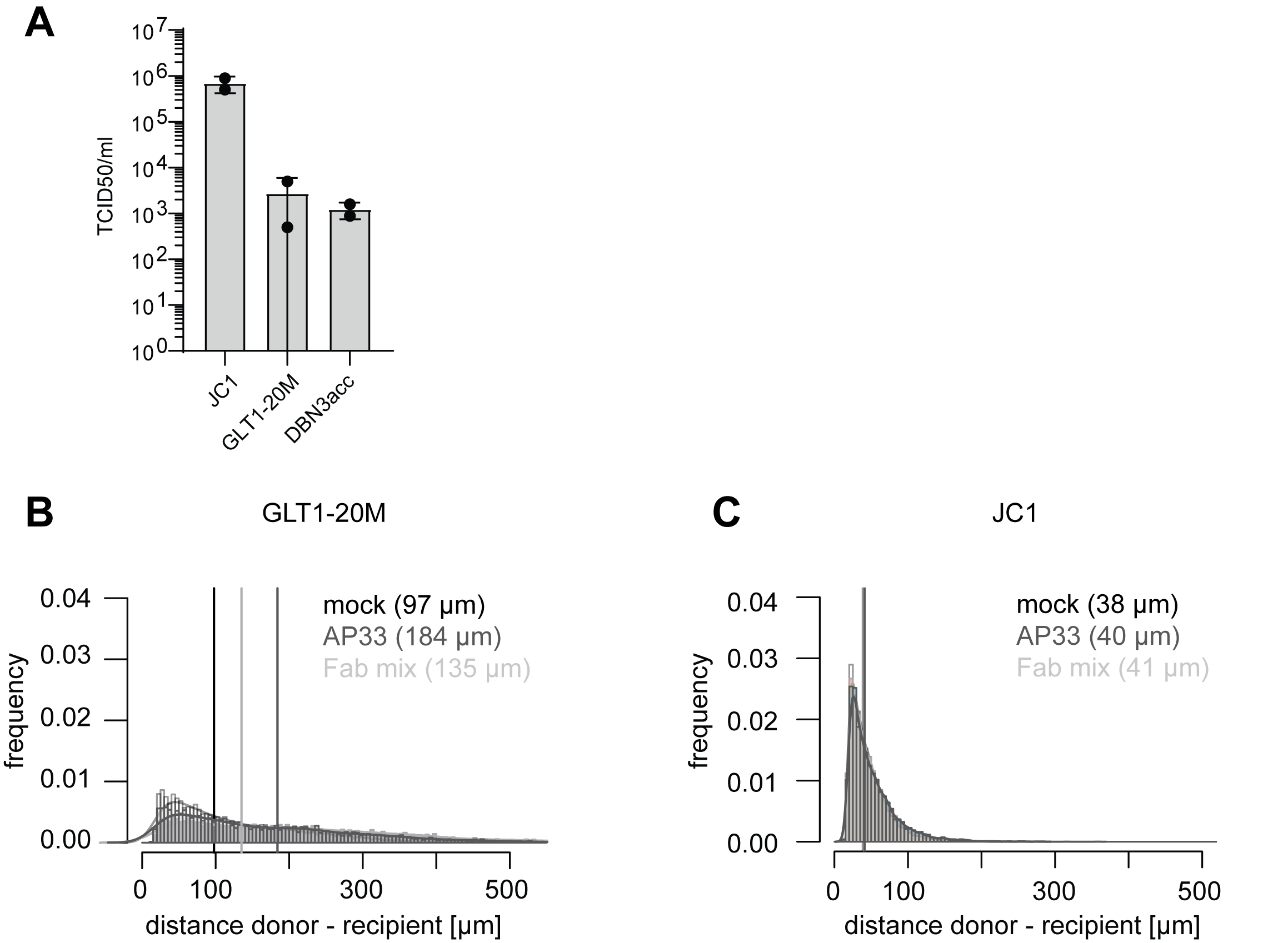

Supplement: S6 Fig — (A) Viral titer (TCID50/ml) from concentrated supernatant of different HCV isolates harvested from Huh7-Lunet CD81 MAVS-GFP-NLS used in Fig 4D in the mock condition. Shown are the mean values and standard deviation of two independent experiments shown as individual data points. (B,C) Distribution of the shortest distances between a GLT1-20M (B) or JC1 (C) positive Huh7-Lunet CD81 MAVS-GFP-NLS cell (“donor”) and the next nearest Huh7-Lunet CD81 MAVS-mCherry-NLS with nuclear mCherry signal (“recipient”) after treatment with HCV neutralizing antibodies or left untreated (mock). Numbers in brackets and vertical lines indicate the median distance for each treatment. Shown are representative data from one experiment (n = 2). (TIF) [file ppat.1010472.s006.tif]

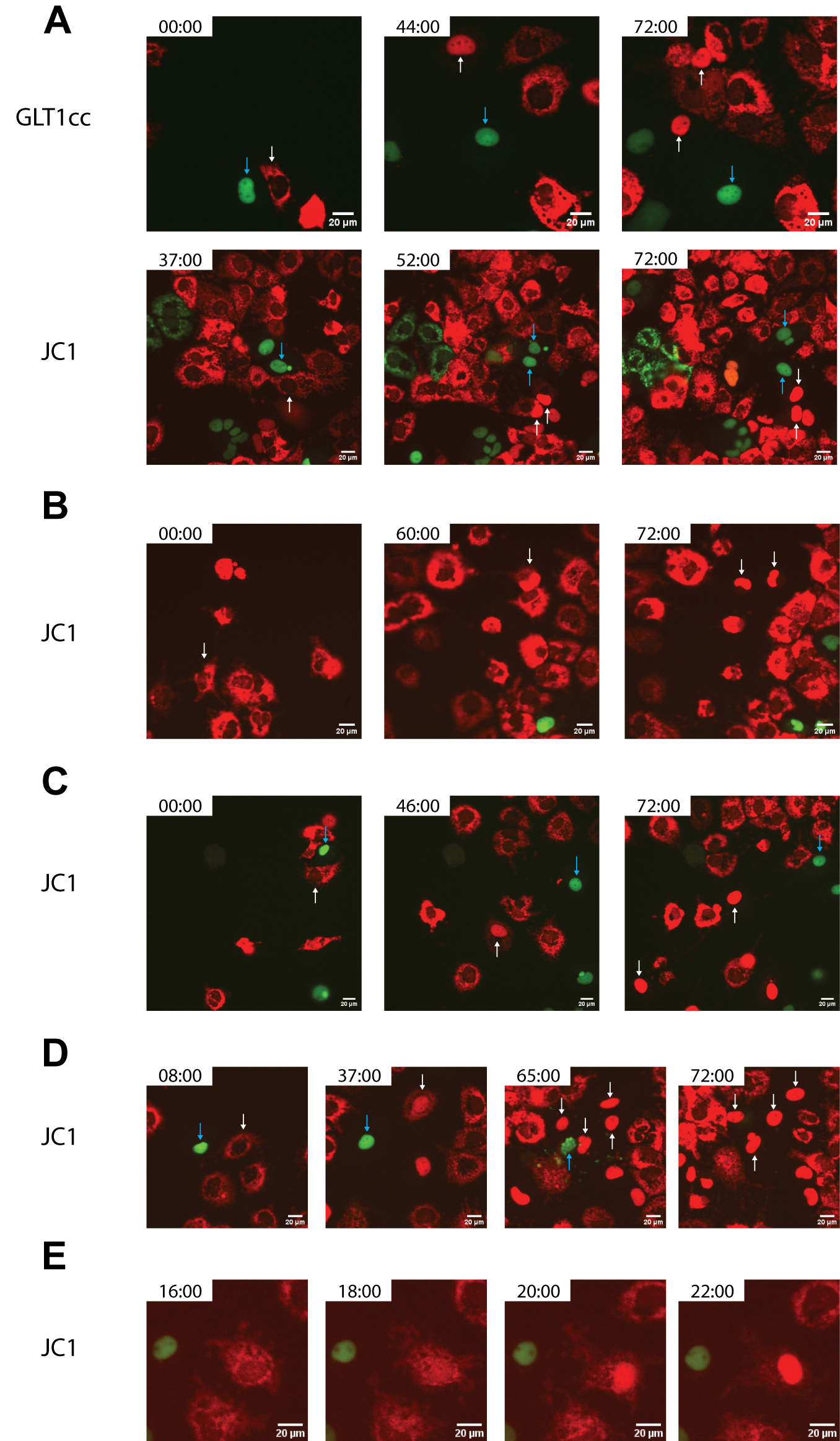

Supplement: S7 Fig — Every 30 minutes an image was acquired for 72 hours. Donor cells of interest are labelled with a cyan arrow, recipient cells of interest are labelled with a white arrow. (A) Example of cell-to-cell mediated spread in both GLT1cc (S1 Movie) and JC1 (S2 Movie). (B-E) Examples based on JC1 because of the overall higher number of infection events. (B) Infection in absence of a visible donor cell representing cell-free spread (S3 Movie). (C) Cell-to-cell spread with recipient cell moving away from donor cell (S4 Movie). (D) Vanishing of donor cell after cell-to-cell spread (S5 Movie). (E) Earliest observed infection event. (TIF) [file ppat.1010472.s007.tif]

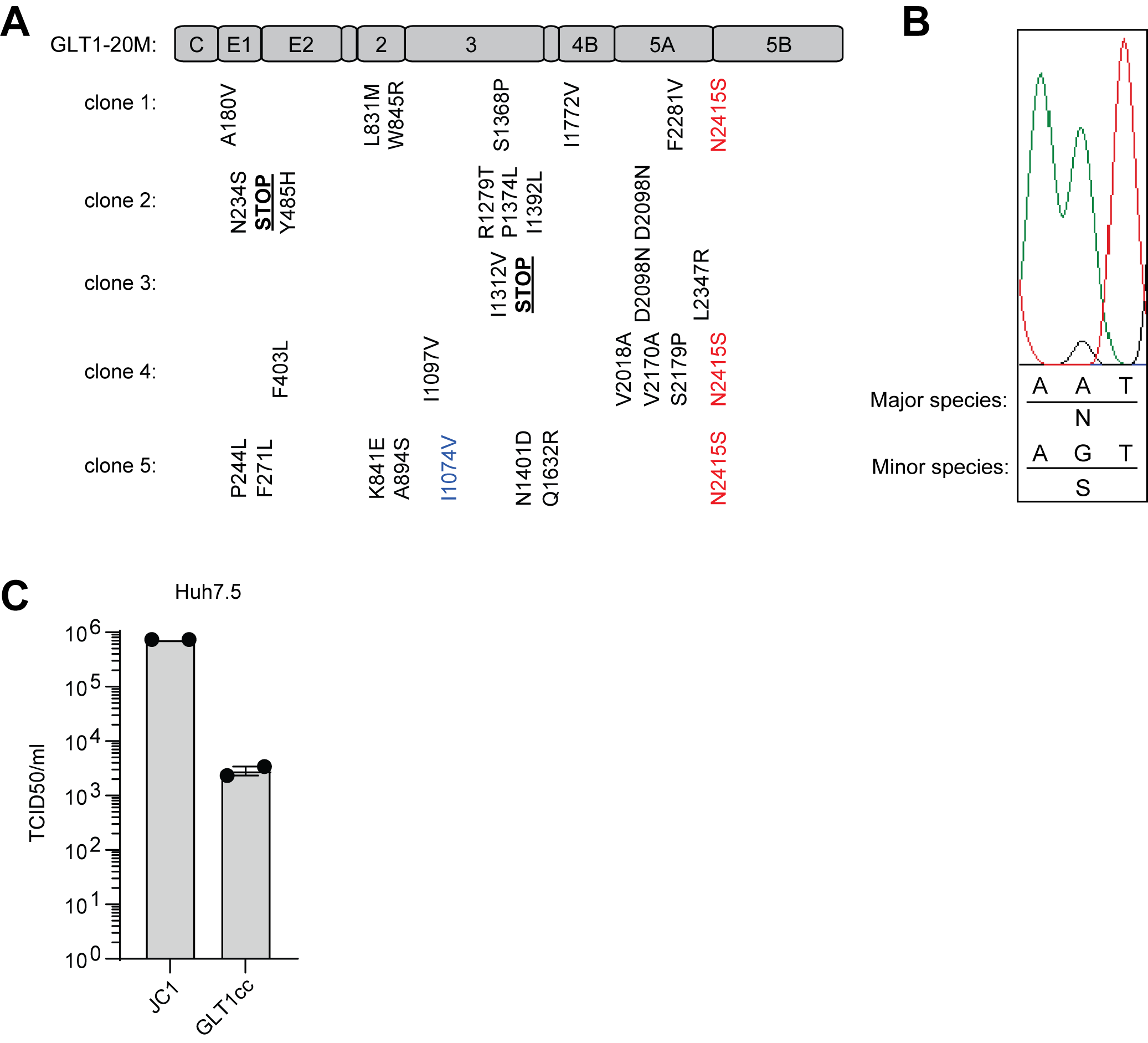

Supplement: S8 Fig — (A) Sequences of 5 individual subclones of RT-PCR products obtained from total RNA of p118.27 compared to GLT1-20M. A missing consensus mutation at position 1074 is shown in blue, premature stop codons are underlined and highlighted in bold. N2415S is shown in red color. (B) Sequence chromatogram of the region encoding N2415S obtained from total RNA of p118.27 by direct sequencing of the amplicon. (C) Viral titer (TCID50/ml) from concentrated supernatant of indicated HCV isolates harvested from Huh7.5 cells; data from two independent biological replicates. (TIF) [file ppat.1010472.s008.tif]

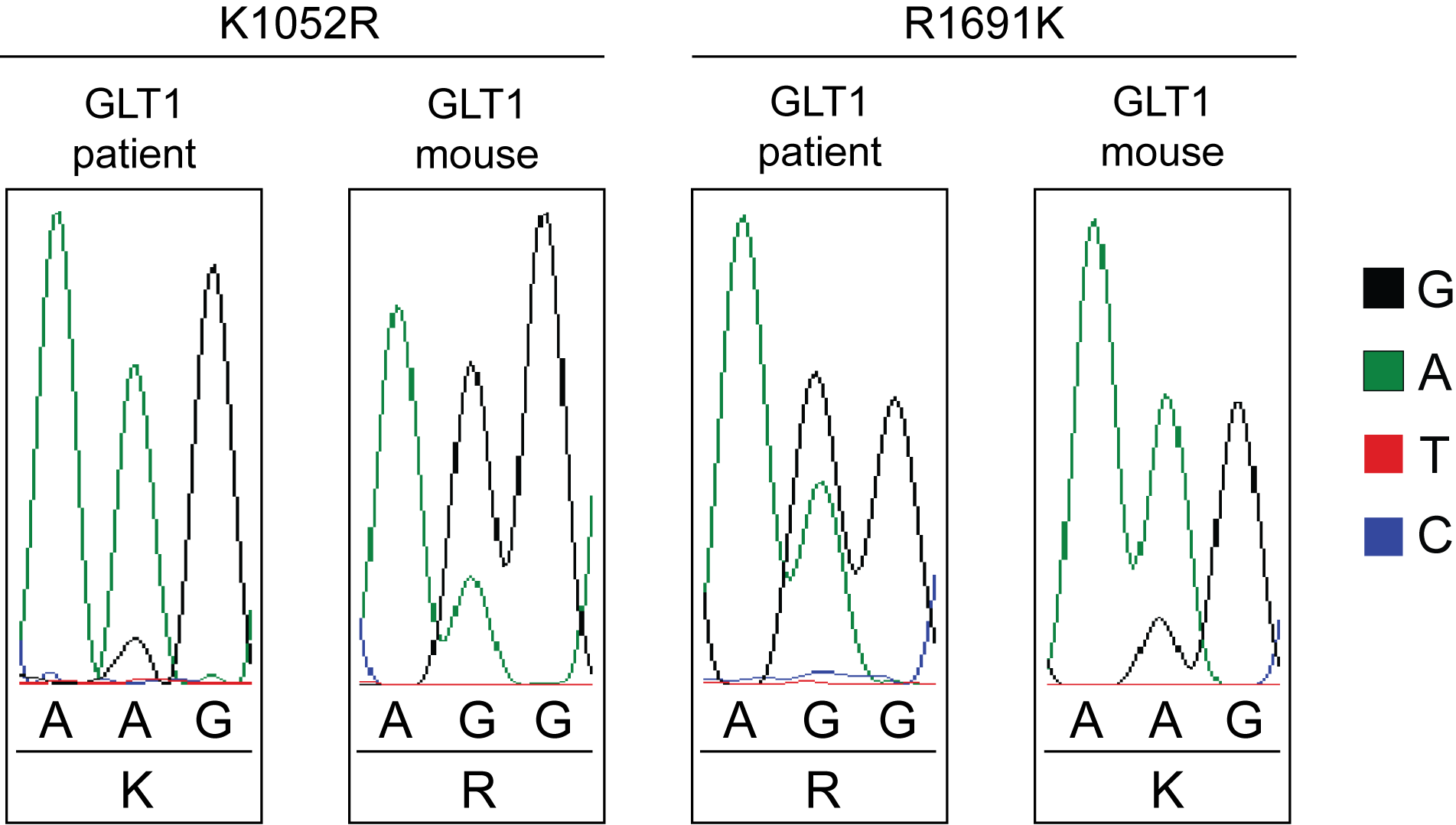

Supplement: S9 Fig — (TIF) [file ppat.1010472.s009.tif]

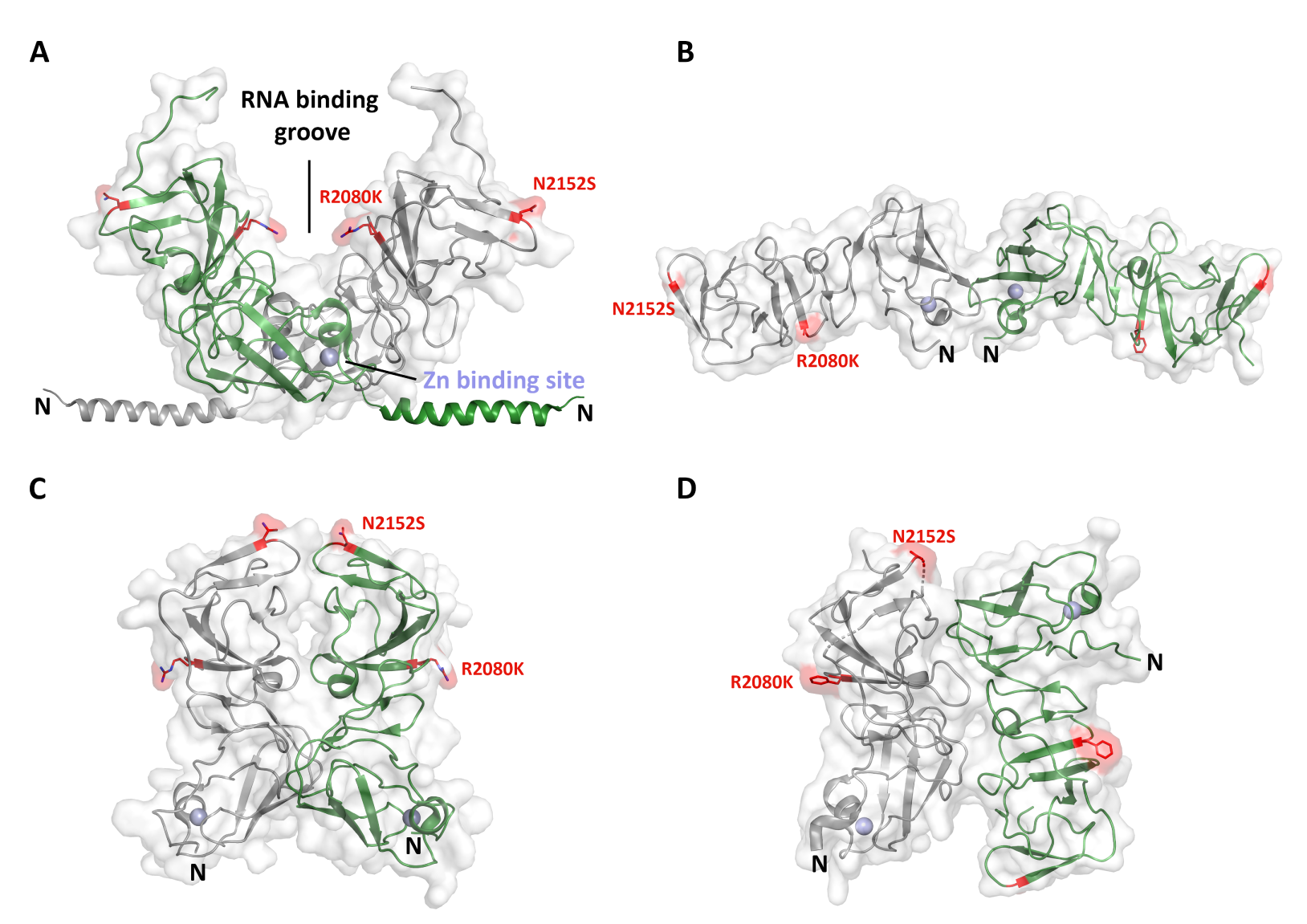

Supplement: S10 Fig — The NS5A domain 1 is anchored to phospholipid membranes by an N-terminal amphipathic helix (residues 1–33, PDB 1R7G). Crystal studies revealed four different dimeric forms of domain 1 from genotype 1a and 1b with the same monomeric unit, but different dimeric arrangements shown. (A) The first crystal structure from genotype 1b (PDB: 1ZH1) with the modelled N-terminal amphipathic helix and the postulated RNA-binding groove between the two monomers is shown. (B) Monomers from genotype 1a (PDB: 4CL1) form a head to head dimer. (C) Genotype 1b monomers assemble in parallel to form an extensive interface (PDB: 3FQM). (D) Monomers from genotype 1a dimerize via a similar interface as shown in C but are assembled in an antiparallel fashion (PDB: 4CL1). (TIF) [file ppat.1010472.s010.tif]
