# Supplementary material for: A Hepatitis C virus genotype 1b post-transplant isolate with high replication efficiency in cell culture and its adaptation to infectious virus production in vitro and in vivo
Source: PLoS Pathog. 2022 Jun 28;18(6):e1010472. doi: 10.1371/journal.ppat.1010472 (PMC9273080; doi:10.1371/journal.ppat.1010472)
Supplement: S1 Text — (DOCX) [file ppat.1010472.s011.docx]

S1 Text. Supplementary methods.

PHH

Primary human hepatocytes were prepared in house at Heidelberg University Hospital. The human liver tissue used to extract PHHs was obtained from a patient undergoing liver cirrhosis which was approved by the French National Ethics Committee and legal instances. No patient information was available in the laboratory. After extraction, PHH were plated in 6-well culture dishes at 1.5x10^6^ cells/well. Protein lysates were prepared 4 days after seeding.

Western blot

For SDS-PAGE/Western blotting, approximately 1×10^6^ cells were lysed in 50 μl lysis buffer (50 mM Tris-HCl, pH 7.4, 150 mM NaCl, 15 mM MgCl_2_, 1% Triton X-100) containing an EDTA-free protease inhibitor cocktail pill (Roche) for 1 h on ice. After centrifugation with 14,000 rpm for 30 min at 4 °C, the cleared supernatant was mixed with 50 μl 2×Laemmli buffer and denatured at 95 °C for 10 min. The lysate of approximately 1×10^5^ cells was loaded in one lane of a polyacrylamide–SDS gel with an appropriate percentage for the protein of interest. The color-prestained protein standard, broad range (11 to 245 kDa) (New England Biolabs), was used to determine the apparent molecular weight of the proteins. After the separation, proteins were transferred to a PVDF membrane using a semi-dry blotter according to the instructions of the manufacturer. The membrane was blocked for 1 h in 5% milk or BSA in 0.5% TBS-Tween20. The primary antibody was diluted in 3% milk-0.5% TBS-T and incubated overnight at 4 °C shaking. After three washing steps with 0.5% TBS-T, the secondary antibody was diluted in 3% BSA or milk in 0.5% TBS-T for 1 h at room temperature. The detection was done using Clarity ECL blotting substrate (Bio-Rad) and the Advanced ECL imaging system (Intas Science Imaging Instruments). The signal intensity was quantified using Fiji.
