# Supplementary material for: A Hepatitis C virus genotype 1b post-transplant isolate with high replication efficiency in cell culture and its adaptation to infectious virus production in vitro and in vivo
Source: PLoS Pathog. 2022 Jun 28;18(6):e1010472. doi: 10.1371/journal.ppat.1010472 (PMC9273080; doi:10.1371/journal.ppat.1010472)
Supplement: S1 Table — (DOCX) [file ppat.1010472.s012.docx]

**Table S1: DNA oligonucleotides used in this study.**

| Oligo Name | Sequence | Use |
| --- | --- | --- |
| A_9416 | CAGGATGGCCTATTGGCCTGGAG | cDNA generation |
| S_59 | TGTCTTCACGCAGAAAGCGTCTAG | cDNA amplification/qPCR |
| A_3580 | GTCCAGCACACGCCATTGAC | cDNA amplification |
| S_93 | AAGCGTCTAGCCATGGCGTT | cDNA amplification |
| A_3489 | GGCCTGTGAGGCTAGTGATGATACA | cDNA amplification |
| S_3317 | GTGCGGGGACATCATCTCGG | cDNA amplification |
| A_6103 | GCTATCAGCCGGTTCATCCACTGC | cDNA amplification |
| S_3420 | TCGCCCATCACGGCCTAC | cDNA amplification |
| A_5982 | TGACCAGGTCCTCGGTGGAG | cDNA amplification |
| S_4540 | GTAACGAGCTCGCCGCGCAGCTGTC | cDNA amplification |
| A_9386 | TTAGCTCCCCGTTCATCGGTTGG | cDNA amplification |
| S_5120 | GTGCGCCAGGGCTCAGGCTCCACC | cDNA amplification |
| A_9364 | GGAGCAGGTAGATGCCTACCCCTAC | cDNA amplification |
| A_9605 | ACATGATCTGCAGAGAGGCCAGTATCAGCAC | cDNA amplification |
| GLT1_probe | 6-FAM–TCCTGGAGGCTGCACGACACTCAT–TAMRA | qPCR |
| A_165 | TACTCACCGGTTCCGCAGA | qPCR |
| Jc1_probe | 6-FAM–AAAGGACCCAGTCTTCCCGGCAATT–TAMRA | qPCR |
| S_146 | TCTGCGGAACCGGTGAGTA | qPCR |
| A_219 | GGGCATAGAGTGGGTTTATCCA | qPCR |
| pFK_bb_fwd | TTCAACGACTCCATGGCCTTAGCGCATTTTC | pFK i341 PiLuc NS3-3´ GLT1-20M, pFK i341 PiLuc NS3-3´ GLT1cc |
| pFK_bb_rev | TGTGAGGCTAGTGATGATACAGCTAAGCATGC | pFK i341 PiLuc NS3-3´ GLT1-20M, pFK i341 PiLuc NS3-3´ GLT1cc |
| GLT1cc_fwd | GTATCATCACTAGCCTCACAGGCCGGGA | pFK i341 PiLuc NS3-3´ GLT1-20M, pFK i341 PiLuc NS3-3´ GLT1cc |
| GLT1cc_rev | AAGGCCATGGAGTCGTTGAATGATCTGAGGTAGGTC | pFK i341 PiLuc NS3-3´ GLT1-20M, pFK i341 PiLuc NS3-3´ GLT1cc |
| S_JCN2a | CTGTGGTGGTTGTGCTATCTCC | pFK i389 GLT1/C3JFH-1 N2A, pFK i389 GLT1cc/C3JFH-1 N2A |
| A_JCN2a | GGGCCCGGGATTTTCCTC | pFK i389 GLT1/C3JFH-1 N2A, pFK i389 GLT1cc/C3JFH-1 N2A |
| S_GLT1_Insert | GAAAATCCCGGGCCCATGAGCACGAATCCTAAACCTC | pFK i389 GLT1/C3JFH-1 N2A, pFK i389 GLT1cc/C3JFH-1 N2A |
| A_GLT1_Insert | GCACAACCACCACAGGAGCTTCGCGAGGAACACTTTATAG | pFK i389 GLT1/C3JFH-1 N2A, pFK i389 GLT1cc/C3JFH-1 N2A |
| S_EcoRI | ACGCATTCTGGCGGAATTCAG | pFK i389 JcN2AΔE1E2 |
| A_NotI | CAGAATATAGTGACGGCCCACG | pFK i389 JcN2AΔE1E2 |
| A_E1 | CTTCTCTAGTGCAGCGGAGACCG | pFK i389 JcN2AΔE1E2 |
| S_E2 | CGGTCTCCGCTGCACTAGAGAAG | pFK i389 JcN2AΔE1E2 |
| S_KpnI | CAAGCTTGGTACCGAGCTCGGATCCATGGACCTCATGGGGTA | pcDNAΔcE1E2-GLT1, pcDNAΔcE1E2-GLT1cc |
| A_XbaI | TAGGGCCCTCTAGATTAGGCCTCAGCCTGGGCT | pcDNAΔcE1E2-GLT1, pcDNAΔcE1E2-GLT1cc |
| S_437_mut | GAACTGGGTTCCTTGCCGC | pFK GLT1cc L437F |
| A_437_mut | GCGGCAAGGAACCCAGTTC | pFK GLT1cc L437F |
| S_ClaI | GTAAGGTTATCGATACCCTCACAT | pFK GLT1cc L437F |
| A_XhoI | CGCTCTCCTCGAGTCCAATTG | pFK GLT1cc L437F |
| S_GLT1_K1846T | TAGGTCTTGGGACGGTGCTTGTGGAC | pFK i341 PiLuc NS3-3´ GLT1 K1846T (mut4B), pFK GLT1-mut4B |
| A_GLT1_K1846T | GTCCACAAGCACCGTCCCAAGACCTA | pFK i341 PiLuc NS3-3´ GLT1 K1846T (mut4B), pFK GLT1-mut4B |
| S_GLT1_S2201R | GCCAGCTCTTCCGCTCGCCAGTTGTC | pFK i341 PiLuc NS3-3´ GLT1 S2201R (mut5A) |
| A_GLT1_S2201R | GACAACTGGCGAGCGGAAGAGCTGGC | pFK i341 PiLuc NS3-3´ GLT1 S2201R (mut5A) |
| S_GLT1_R2884G | CATTCAAGGACTCCATGGCCTTAG | pFK i341 PiLuc NS3-3´ GLT1 R2884G (mut5B) |
| A_GLT1_R2884G | CTAAGGCCATGGAGTCCTTGAATG | pFK i341 PiLuc NS3-3´ GLT1 R2884G (mut5B) |
| S_GLT1_NS5A_mcherry | CAAGCGGTCCGAAGGGGAGCCG | pTM NS3-5B GLT1-NS5A_mCherry |
| A_GLT1_NS5A_mcherry | CAAGCGGTCCGAAGGGGAGCCGG | pTM NS3-5B GLT1-NS5A_mCherry |
| S_N2415S | GGTGATAGTGTAGTCTGCTGCTC | pFK-GLT1-mut4B+N2415S, pFK-GLT1-20M+N2415S (GLT1-21M/GLT1cc) |
| A_N2415S | GAGCAGCAGACTACACTATCACC | pFK-GLT1-mut4B+N2415S, pFK-GLT1-20M+N2415S (GLT1-21M/GLT1cc) |
| S_1905 | TTCGGCGTCCCTACGTACAGTTG | pFK Con1/C3GLT1 |
| A_Con1_MMC3 | CTCAGCTCTGGTGATAAGATACTGTAACCACCATATGAGCCTAGCGAG | pFK Con1/C3GLT1 |
| S_MMC3 | CAGTATCTTATCACCAGAGCTGAGG | pFK Con1/C3GLT1 |
| A_3254 | AACTGCCACCGCAAGGTCTCGTAG | pFK Con1/C3GLT1 |
