# Supplementary material for: A Hepatitis C virus genotype 1b post-transplant isolate with high replication efficiency in cell culture and its adaptation to infectious virus production in vitro and in vivo
Source: PLoS Pathog. 2022 Jun 28;18(6):e1010472. doi: 10.1371/journal.ppat.1010472 (PMC9273080; doi:10.1371/journal.ppat.1010472)
Supplement: Attachment — Submitted filename: Comments.docx [file ppat.1010472.s018.docx]

In this terrific manuscript, the authors report an infectious cDNA clone of HCV genotype 1b and perform its detailed in vitro and in vivo characterization. The cDNA clone, referred to as GLT1cc, was derived from a cell culture-adapted version of an HCV consensus sequence recovered from the serum of a liver transplant patient. The authors began by constructing subgenomic and full-length replicons from the wild-type consensus sequence and testing their replication capacity in human hepatoma cells under various replication-enhancing conditions. While the subgenomic replicon replicated to high levels, the full-length replicon failed to replicate. The authors then performed a systematic and meticulous effort of passaging the full-length replicon in cultured cells for over one year, ultimately obtaining a cell culture-adapted version that yielded high levels of replication and produced infectious virus particles, which spread in culture in E2 and CD81-depenent manner. When injected into humanized liver mice, the animals developed viremia, indicating that the virus recovered from the GLT1cc molecular clone was capable of infecting human liver cells.

Overall, the hypotheses are well formulated, the experiments are nicely designed, and the results are presented in a comprehensible manner. The study fills an important gap in the field by providing an efficient cell culture system for HCV genotype 1b that can be used, along with other genotypes, to investigate mechanisms underlying HCV pathogenesis and to test potential vaccine and therapeutic candidates. I therefore strongly support the publication of this work in PLoS Pathogens. There are however a few concerns that should be addressed before making the final decision.

**Major points**

1. All experiments demonstrating HCV spread in cell culture are based on nuclear translocation of a fluorescent protein from the mitochondrial membrane to the nucleus. Using this system, the authors made an observation that GLT1 spread faster in culture despite low amounts of free virus in the supernatant. This observation could not be attributed to higher cell-to-cell transmission of GLT1 (GLT1 cell-to-cell spread was not any better than that of JFH-1). One possibility that can explain this phenotype is the higher proteolytic efficiency of GLT1 NS3/4A protease, leading to higher nuclear translocation of the fluorescent signal. The authors should test, or at the very least, discuss this possibility.
2. A significant portion of the manuscript has been dedicated to analyzing the difference between the molecular clones GLT1-20M and GLT1cc, which differ from each other by one amino acid. According to the authors, GLT1-20M was established from the HCV consensus sequence after 118 viral passages in cell culture. If this is the case, it is unclear as to why N2415S was not included in GLT1-20M, when this mutation was identified in three out of the five tested clones (p. 15, lines 366-367). Shouldn’t a mutation present in three out of five clones be considered a consensus mutation? Please clarify.
3. P. 9, lines 223-226. The authors suggest that the reduced activation of PI4KA by GLT1, as compared to Con1, is the potential reason for increased replication of GLT1 in HuH-7 cells. Figure 1C shows that the combined inhibition of PI4KA and CKIα causes much stronger increase in GLT1 replication compared to Con1. Combining these two observations, it is clear that GLT1 replication in itself inhibits PI4KA activation and further benefits from chemical inhibition of PI4KA. Does this mean that GLT1 is exceptionally sensitive to PI4KA? Please discuss.
4. The rationale and lessons learnt from the single-cell analysis of viral spread in cell culture (p.14-15, lines 339-357) are unclear to this reviewer. Also, the logic behind choosing 50 µM radius is not described. Is the presence of recipient cells within 50 µM radius of a donor cell indicative of both cell free and cell-to-cell spread? The fact that addition of neutralizing antibodies had only a minimal effect on the number of recipient cells in Fig. 4G argues in favor of cell-to-cell spread being the major mode of transmission in this system. Please clarify these points in the manuscript.
5. P. 13, lines 317-318. The authors suggest that GLT1-20M spread in cell culture with nearly the same efficiency and kinetics as JC1. This does not seem to be the case in Fig. 4B, where JC1 yielded almost double the number of positive cells as GLT1-20M at 72h post-infection. The difference of 2-fold can be quite significant in this case. If the authors want to describe these results with reference to the amount of virus generated by infected cells, they should normalize the number of positive cells against viral titers and present these values as a separate graph.
6. Please apply statistical analysis to all figures, mainly Fig. 4E, F, G and Fig. 6B, to determine if subtle differences shown in these figures are statistically significant.

**Minor points**

1. P. 3, lines 73-75. The sentence seems to be incomplete. It appears to be a “not only, but also” sentence, but with the “but also” part missing.
2. The patient serum used in this manuscript appears to have been described in the previous report published by the authors. If this is the case, the authors should describe how this serum sample was defined in the previous publication. This will allow the readers to track the history of the serum sample.
3. P. 11, lines 254-255. The authors state that no infection events were detectable from supernatants of GLT1 at 72 h after transfection, and they refer to Fig. 3B. However, the data for GLT1 has not been shown in this figure. The authors should add a panel to show GLT1-infected cells.
4. P. 16, line 379. The sentence is incomplete.
