# Supplementary material for: A Hepatitis C virus genotype 1b post-transplant isolate with high replication efficiency in cell culture and its adaptation to infectious virus production in vitro and in vivo
Source: PLoS Pathog. 2022 Jun 28;18(6):e1010472. doi: 10.1371/journal.ppat.1010472 (PMC9273080; doi:10.1371/journal.ppat.1010472)
Supplement: Attachment — Submitted filename: ResponseToReviewers_GLT1.docx [file ppat.1010472.s019.docx]

**Point by point response to reviewer comments**

**Reviewer #1:**

*In this terrific manuscript, the authors report an infectious cDNA clone of HCV genotype 1b and perform its detailed in vitro and in vivo characterization. The cDNA clone, referred to as GLT1cc, was derived from a cell culture-adapted version of an HCV consensus sequence recovered from the serum of a liver transplant patient. The authors began by constructing subgenomic and full-length replicons from the wild-type consensus sequence and testing their replication capacity in human hepatoma cells under various replication-enhancing conditions. While the subgenomic replicon replicated to high levels, the full-length replicon failed to replicate. The authors then performed a systematic and meticulous effort of passaging the full-length replicon in cultured cells for over one year, ultimately obtaining a cell culture-adapted version that yielded high levels of replication and produced infectious virus particles, which spread in culture in E2 and CD81-depenent manner. When injected into humanized liver mice, the animals developed viremia, indicating that the virus recovered from the GLT1cc molecular clone was capable of infecting human liver cells.*

*Overall, the hypotheses are well formulated, the experiments are nicely designed, and the results are presented in a comprehensible manner. The study fills an important gap in the field by providing an efficient cell culture system for HCV genotype 1b that can be used, along with other genotypes, to investigate mechanisms underlying HCV pathogenesis and to test potential vaccine and therapeutic candidates. I therefore strongly support the publication of this work in PLoS Pathogens. There are however a few concerns that should be addressed before making the final decision.*

We are very pleased by the positive assessment of our manuscript by this reviewer!

**Major points**

1. *All experiments demonstrating HCV spread* *are based on nuclear translocation of a fluorescent protein from the mitochondrial membrane to the nucleus in cell culture. Using this system, the authors made an observation that GLT1 spread faster in culture despite low amounts of free virus in the supernatant. This observation could not be attributed to higher cell-to-cell transmission of GLT1 (GLT1 cell-to-cell spread was not any better than that of JFH-1). One possibility that can explain this phenotype is the higher proteolytic efficiency of GLT1 NS3/4A protease, leading to higher nuclear translocation of the fluorescent signal. The authors should test, or at the very least, discuss this possibility.*

The reviewer is right in bringing up this possibility. We overall favor the hypothesis that cell-to-cell transmission is the preferred transmission mode of all HCV isolates, at least in cell culture. Although it has the same requirements regarding virion morphogenesis as cell free spread, it is more frequent and easier to detect than cell free virus for unknown reasons. And we indeed do find an increased cell-to-cell spread efficiency of GLT1 during passaging, in line with slowly increasing TCID50 titers (Fig. 3B and 4A and D). In addition, we found only one mutation in the protease gene (V1074I), which is common among gt1b isolates (Fig. 6A) and none in NS4A, further rendering the possibility of increased protease activity unlikely. We therefore decided to discuss this option, as suggested by the reviewer, and added a sentence to the discussion, addressing this important possibility.

2. *A significant portion of the manuscript has been dedicated to analyzing the difference between the molecular clones GLT1-20M and GLT1cc, which differ from each other by one amino acid. According to the authors, GLT1-20M was established from the HCV consensus sequence after 118 viral passages in cell culture. If this is the case, it is unclear as to why N2415S was not included in GLT1-20M, when this mutation was identified in three out of the five tested clones (p. 15, lines 366-367). Shouldn’t a mutation present in three out of five clones be considered a consensus mutation? Please clarify.*

The consensus sequence of GLT1-20M was obtained by direct sequencing of a PCR product. Here, the sequence chromatogram showed a signal intensity of 526 for the variant encoding N, compared to 52 for S (ca. 10%). N2415S was therefore not identified as a major variant. The individually sequenced plasmid clones were generated from an independent RT-PCR reaction from the same RNA-preparation, here 3 out of 5 clones (60%) showed the mutation. We have no explanation for this discrepancy, but it was most likely due to a stochastic variation either in the PCR reaction or the cloning procedure. We have added this information to the results section and added Fig. S8B, showing the respective chromatogram of the amplicon sequencing.

3. *P. 9, lines 223-226. The authors suggest that the reduced activation of PI4KA by GLT1, as compared to Con1, is the potential reason for increased replication of GLT1 in HuH-7 cells. Figure 1C shows that the combined inhibition of PI4KA and CKIα causes much stronger increase in GLT1 replication compared to Con1. Combining these two observations, it is clear that GLT1 replication in itself inhibits PI4KA activation and further benefits from chemical inhibition of PI4KA. Does this mean that GLT1 is exceptionally sensitive to PI4KA? Please discuss.*

We thank this reviewer for bringing up this interesting question. Based on the fact that all replication stimulating conditions have comparable effects on different gt1b isolates, we assume that all of them represent the intrinsic replication capacity of different isolates (see discussion). Using naïve Huh7 cells, gt1b wt replication is barely detectable, but still a bit higher for GLT1 compared to Con1 (Fig. 1C, black lines). Since this phenotype correlates with reduced PI4KA activation (now shown in Fig. 2D/E) and reduced DMV size (Fig. S4B), the same phenotypes underlying the mechanism of some replication enhancing mutations, it is tempting to speculate that the reduced PI4KA activation is the cause of the slightly enhanced replication of GLT1 wt in naïve Huh7. However, the reduction of PI4KA activity upon PCi treatment is far more pronounced (compare DMSO/Mock with 5 µM H479/1 µM PI4KA-A in reviewer Fig. 1). We therefore assume that the slightly reduced PI4KA induction of GLT1 wt compared to Con1 wt is rather marginal, in line with the moderate impact on replication, and that the effect of PCi treatment is simply additive. An only moderate impairment of PI4KA activation is also a prerequisite for efficient replication of GLT1 wt in vivo, since titer enhancing mutations abrogating PI4KA activation have been found to interfere with replication in Chimpanzees. We have added this important aspect to the discussion section.

Reviewer Fig. 1: Impact of PCi treatment on PI4P accumulation. Cells expressing NS3-5B (Con1 wt) were treated with various concentrations of H479 and a PI4KA inhibitor, the individual components of PCi, and PI4P induction was assessed as in Fig. 2 of our manuscript. The arrowhead points to the currently used PCi treatment conditions (Fig. 5c, taken from Harak et al. Tuning a cellular lipid kinase activity adapts hepatitis C virus to replication in cell culture. Nat Microbiol. 2016;2:16247.)

4. *The rationale and lessons learnt from the single-cell analysis of viral spread in cell culture (p.14-15, lines 339-357) are unclear to this reviewer. Also, the logic behind choosing 50 µM radius is not described. Is the presence of recipient cells within 50 µM radius of a donor cell indicative of both cell free and cell-to-cell spread? The fact that addition of neutralizing antibodies had only a minimal effect on the number of recipient cells in Fig. 4G argues in favor of cell-to-cell spread being the major mode of transmission in this system. Please clarify these points in the manuscript.*

The analysis at single-cell resolution was intended to evaluate the efficiency of viral spread at a local level, i.e. determining how efficient individual donor cells spread the infection to neighboring cells. Without the ability to identify membrane contacts within the image analysis and cell localization defined by the position of the nuclei, cells were defined as being in contact if the distance between two nuclei was smaller than a predefined radius. The rationale for choosing a radius of r=50 µM was given in Materials and Methods and was based on the assumption that cells had an average diameter of 20-30 µM. With this assumption, cells had on average 5 direct neighbors, as could be expected within dense cell cultures. We also performed our analyses using smaller and larger radii to define local cell areas (r=30 and 100 µM), which did not change our results.

Indeed, the minimal effect of the neutralizing antibodies on the frequency of donor foci that are able to spread in JC1 (Figure 4G) could argue for cell-to-cell spread as the major mode of viral transmission. However, due to the indicated limitations that affect the determination of cell contact events (motility of donor and recipient cells, time between transmission and nuclear transfer), we think that it is currently difficult to make clear propositions about the contribution of the different viral transmission modes for JC1 and GLT1-20M. A more detailed analysis involving detailed dynamic models considering cell motility and delayed fluorescence would be needed to address this point appropriately.

We revised the corresponding paragraphs within the manuscript to clarify all these points.

5. *P. 13, lines 317-318. The authors suggest that GLT1-20M spread in cell culture with nearly the same efficiency and kinetics as JC1. This does not seem to be the case in Fig. 4B, where JC1 yielded almost double the number of positive cells as GLT1-20M at 72h post-infection. The difference of 2-fold can be quite significant in this case. If the authors want to describe these results with reference to the amount of virus generated by infected cells, they should normalize the number of positive cells against viral titers and present these values as a separate graph.*

We thank this reviewer for the great suggestion to think about normalization of the spreading data. However, after careful consideration, we felt that it would further increase the level of complexity of these already complex datasets. On the other end, we fully agree, that a twofold difference observed for JC1 indeed should not be neglected. We therefore decided to tone down the statement in the results by mentioning the clear twofold difference.

6. *Please apply statistical analysis to all figures, mainly Fig. 4E, F, G and Fig. 6B, to determine if subtle differences shown in these figures are statistically significant.*

We apologize for not having performed these tests in the original submission. The differences shown in Fig. 4 indeed were statistically significant, the data in Fig. 6B not. We added this important information to the results section and to the respective figure legends.

**Minor points**

1. *P. 3, lines 73-75. The sentence seems to be incomplete. It appears to be a “not only, but also” sentence, but with the “but also” part missing.*

We have deleted “not only” and the subsequent sentence, following the suggestion of the second reviewer, and think the statement is indeed clearer now.

2. The patient serum used in this manuscript appears to have been described in the previous report published by the authors. If this is the case, the authors should describe how this serum sample was defined in the previous publication. This will allow the readers to track the history of the serum sample.

We fully agree, that this information is helpful for the reader. It is “Serum 1” and we have added this information to the “results” and “methods” section (patient information).

3. P. 11, lines 254-255. The authors state that no infection events were detectable from supernatants of GLT1 at 72 h after transfection, and they refer to Fig. 3B. However, the data for GLT1 has not been shown in this figure. The authors should add a panel to show GLT1-infected cells.

We followed the advice of the reviewer and added panels showing the lack of infected cells for GLT1 in Fig. S5A.

4. P. 16, line 379. The sentence is incomplete.

We apologize and modified the sentence accordingly.

**Reviewer #2:**

*The study by Heuss et al. (PPATHOGENS-D-22-0053), describes a novel cloned hepatitis C genotype 1b strain with intrinsic high levels of replication in cell culture. The authors provide evidence for the mechanism behind this unique phenotype. Through a remarkable process of culture adaptation, the authors then succeed in selecting mutations that overcome a restriction on infectious particle production and perform exhaustive mechanistic analysis. Finally, experiments in human liver chimeric mice show that the cell culture adapted clone retains viability in vivo, and importantly identify a critical mutation in NS5A. Thus, this is the first paper to demonstrate in vivo viability of a highly adapted HCVcc; most existing full-length culture systems depend on a large number of adaptive mutations.
This work is original, highly relevant and represents a significant advance in the field, since most HCV isolates are non-replicative in vitro and genotype 1b, the most prevalent genotype worldwide, have been notoriously difficult to culture. Further, it has important in vivo data. However, there are a few issues that needs to be addressed.*

We are delighted about the very positive general comments on our manuscript by this reviewer.

Specific comments.
*1. The authors should acknowledge/cite the previous work by Jinqian Li et al., who developed genotype 1b cell culture systems (https://doi.org/10.1016/j.antiviral.2021.105136), and therefore tone down or delete statements referencing GLT1cc as the first efficient infectious cell culture system for genotype 1b throughout the text, including in the abstract.*

We deeply apologize for having missed this important paper. This was indeed not on purpose and we are very grateful that this reviewer brought up this point. Besides removing all claims towards GLT1cc being the “first efficient” gt1b infectious cell culture model, we further included some aspects of this and the previous study of this group on the adaptation of a chimeric gt1b-JFH1 isolate into the discussion, due to the striking similarities of some of the observed changes.

*2. The following statement from lines 75-77: “It also provides novel perspectives towards our understanding how liver transplantation drives the evolution of viral isolates with high replication capacity, which might contribute to direct pathogenesis of HCV infection.”
Although certainly a very relevant topic, the authors should tone down the contribution of the study to the understanding of evolution of viruses and liver transplantation, as none of the experiments and data presented address this aspect.*

This is indeed true, we therefore deleted the entire statement.

*3. Were the experimental conditions applied in this study identical to those from references #17 and #30? What is the variability of methods assessing the size of DMVs? In other words, can the authors reliably compare the size of GLT1 DMVs with data on Con1 and JFH1 obtained from previous studies? If not, DMVs of transfected cells with Con1, JFH1 and GLT1 replicons should be measured head-to-head.*

We thank this reviewer for the insightful comment. Indeed, several parameters were changed compared to previous studies, potentially impacting the DMV diameter. This includes the use of correlative light-and-electron microscopy, requiring adjustments in the protocol, as well as the use of NS3-5B expression constructs with an autofluorescent protein encoded in NS5A and a semi-automated diameter analysis, allowing to include far higher DMV numbers. To ensure that these changes did not impact the comparison to previous studies, we included new data in Fig. S4B, based on NS3-5B NS5A_mCherry expression of Con1, since only the expression model allows the unbiased comparison of gt1b wt in the absence of other conditions with potential impact on DMV size (e.g. PCi treatment or adaptive mutations). In fact, we obtained almost identical data for Con1 as in our previous study and therefore are convinced that our new data can be compared to the previous studies. We still mention the potential limitations of comparability in the revised manuscript.

*4. Lines 313-315, the origin of the GLT1-mut4B sample is missing.*

Sorry for lacking this information, we have added it to the results section.

*5. In Fig 4C, can the residual level of Jc1 spread in CD81 deficient cells be explained?*

Thanks for bringing up this point. We did not further follow up this phenotype, since a previous study by Arvind Patel’s lab has used the same CD81-deficient cells and reported on CD81 independent spread, even far more pronounced than in our study. Since we did not further analyze this phenotype, we added this information and the respective citation to the results section.

*6. Lines 323-325: “comparing GLT1-20M with JC1 324 and the cell culture adapted gt3a isolate DBN3acc (36), generating similar titers as GLT1-20M (Fig. S6A).”
Could the authors clarify in which conditions were the titers of GLT1-20M and DBN3acc comparable? In Fig.S6A the titers of GLT1-20M present high variability in case that the depicted bar is indeed an error bar, which should be stated in the figure legend.*

Sorry for not being clearer here. Indeed, titers are comparable under mock conditions (absence of neutralizing antibodies). We have added this information to the results section. And yes, the bars represent error bars, now stated in the respective figure legend.

*7. In the legend of figure 4D, please specify time point measured.*

The time point was 72h pT and has been added.

*8. In figure 4E, correct the typo, both cell lines have the same name.*

We do not exactly understand what the reviewer is referring to here, but we assume it is the use of CD81 vs CD81N cells in the y-axis labelling of Fig. 4B and C. Since the term CD81N has been used for this cell line, lacking CD81 expression, in previous studies, we tried to make the difference clearer by introducing “CD81N” to the figure legend, which so far lacked the term.

*9. Abstract: “but cell-to-cell spreading efficiency was not higher as in other isolates like JFH-1” Suboptimal language – rewrite.*

We fully agree and have rephrased the sentence.

*10. Introduction: “Meanwhile, additional genomes capable of infectious virus production based on gt1a, gt2, gt3a and gt6a have been generated (reviewed in (26)).”
Update to include gt4a, which were recently published also. Subtypes not specified for gt2 (2a, 2b and 2c have been cultured)*

Again our apologies (see point 1). We have added the missing information on gt4a and specified the gt2 subtypes. Of course, we now also introduce the gt1bcc systems.

*11. Introduction: “GLT1cc therefore closes an important gap in the availability of full-replication cycle models of all major HCV genotypes”
Since there is already published full-length culture systems for at least 3 gt1a and 2 gt1b isolates, this statement should be changed to reflect this fact.*

We agree that this statement is not appropriate and deleted it.

*12. Results: “We therefore PCR-amplified the viral genomic sequences from infected Huh7 cells and generated a consensus sequence based on direct sequencing of the PCR products”
The authors should specify here that the 5’ and 3’ terminal sequences were derived from Con1 (and not determined from the patient sequence).*

Indeed, the reviewer is right. We added the missing information (1-82 of the 5’UTR and 31 3’terminal nucleotides) to the results section.

*13. Discussion: “So far they have been established for gt1a, gt2a, gt2b, gt3a and gt6a, with varying efficiency, requiring up to 20 mutations for efficient virus production (reviewed in (26)), but no efficient gt1b isolate is available.”
Update to reflect comments above about HCVcc for genotype 4a and other genotype 1b isolates.*

Again, our apologies. We have modified the discussion as suggested and in addition discussed mutations that appear to be common for gt1cc variants in the light of the gt1bcc papers we previously had missed.
